# Intramuscular Midazolam for Pediatric Sedation in the Emergency Department: A Short Communication on Clinical Safety and Effectiveness

**DOI:** 10.5812/traumamon.3458

**Published:** 2012-05-26

**Authors:** Mohammad Reza Ghane, Seyed Yasin Musavi Vaezi, Amir Abbas Hedayati Asl, Hamid Reza Javadzadeh, Sadrollah Mahmoudi, Amin Saburi

**Affiliations:** 1 Trauma Research Center, Baqiyatallah University of Medical Sciences, Tehran, IR Iran; 2 Pediatric Department, Baqiyatallah University of Medical Sciences, Tehran, IR Iran; 3 Chemical Injury Research Center, Baqiyatallah University of Medical Sciences, Tehran, IR Iran

**Keywords:** Injections, Intramuscular, Midazolam, Safety, Sedation, Euphoria

## Abstract

**Background:**

Procedural sedation in children continues to be a problem in the emergency department (ED). Midazolam is the first water-soluble benzodiazepine and it has been widely used for procedural sedation in pediatric patients.

**Objectives:**

The aim of this study was evaluation of clinical safety and effectiveness of intramuscular Midazolam for pediatric sedation in the ED setting.

**Materials and Methods:**

We performed a self-controlled clinical trial on 30 children who referred to the Baqiyatallah Hospital ED between 2009 and 2010. They received intramuscular Midazolam 0.3 mg/kg for procedural sedation and then they were followed for sedative effectiveness and safety. Vital signs and O2 saturation were also observed. The findings were compared using SPSS ver. 16 software.

**Results:**

The mean age was 5.50 ± 2.70 years, the mean weight was 19.50 ± 6.63 kilograms and 16 patients (53.3%) were females. The most common adverse effect was euphoria (66.66%) and vertigo (6.7%); 27.7% did not show any side effects. There was an overall complication rate of 72.3%. The vital signs including heart rate, respiratory rate, systolic and diastolic blood pressure and O2 saturation decreased significantly during sedation (P value < 0.05).

**Conclusions:**

Midazolam is an effective and relatively safe sedative for pediatric patients in the ED. The patient should be observed closely and monitored for psychological and hemodynamic side effects.

## 1. Background

Procedural sedation in children continues to be a problem in the emergency department (ED). Midazolam is a benzodiazepine that has been widely used for procedural sedation in adults (1). Various sedatives such as pentobarbital, propofol, fentanyl, ketamine and methohexital have been suggested for pediatric sedation but it seems that the selection of sedative agents was based on preference(2). The literature has little documentation on midazolam safety and efficacy in pediatric emergency departments; but there is an increasing interest to use midazolam for pediatric sedation and analgesia(3). We used intra-muscular (IM) midazolam to provide sedation for imaging in ED and then evaluated the efficacy and safety of midazolam for sedation and anxiety of children in the ED.

## 2. Objectives

The aim of this study was evaluation of clinical safety and effectiveness of intramuscular midazolam for pediatric sedation.

## 3. Materials and Methods

We conducted a before-after clinical trial on a highly selective group of 30 children between 2 and 12 years-old. The children who presented to the ED of the Baqiyatallah Hospital between 2009 and 2010 were enrolled. The patients that met the inclusion criteria received intramuscular midazolam 0.3 mg/kg, before imaging (CT-Scan or magnetic resonance imaging). Midazolam was administrated at least 30 minutes before beginning the procedure. Sedation, irritability and cooperation scores were followed every 15 minutes during the first hour after receiving the drug. Five stages for sedation were assessed .(4).

Also, the vital signs and O_2_ saturation were observed during the sedation. The findings were analyzed by using t-test, Chi-square and repeated measure ANOVA SPSS ver. 16; and P value < 0.05 was considered statistically significant. This study was approved by ethics committee of our university and the parents filled an informed consent before enrollment.

## 4. Results

**Figure 1.**
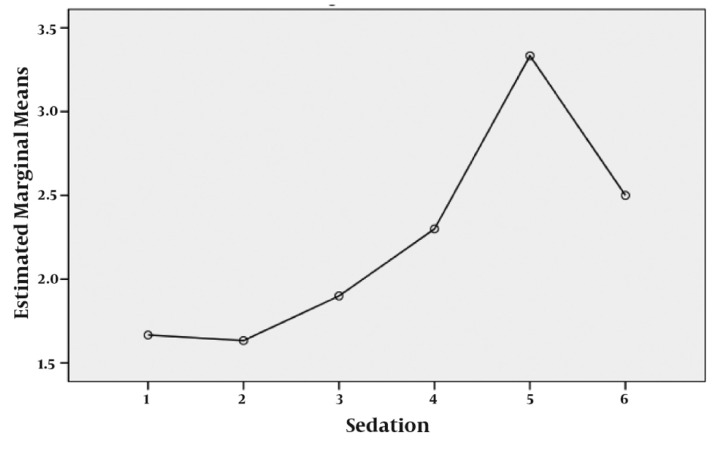
Changes in Sedation Score before, 5 minute, 15, 30, 45 and 60 minutes after injection.

The mean age was 5.50 ± 2.70 years, the mean weight was 19.50 ± 6.63 Kg and 16 patients (53.3%) were female. All of the patients were sedated completely after the first dose. The trend of sedation staging progressed to deep sleep; irritability progressed to complete calmness (Figure 1 & Table 1). These trends were statistically significant (P value < 0.001).

**Table 1.** Changes in sedation, irritability and cooperation score before, and 5, 15, 30, 45 and 60 minutes after injection.

	Sedation					Irritability				Cooperation		
Score	1	2	3	4	5	1	2	3	4	1	2	3
Before prescription, %	60	20	16.6	0	3.4	20	53.3	26.6	0	80	10	10
5 Min after prescription, %	60	23.3	13.3	0	3.4	16.6	56.6	26.6	0	76.6	13.3	10
10 Min after prescription, %	40	36.6	20	0	3.4	13.3	50	36.6	0	56.6	30	13.3
15 Min after prescription, %	23.3	33.3	36.6	3.4	3.4	6.6	16.6	73.3	3.4	30	40	30
30 Min after prescription, %	10	10	30	36.6	13.3	13.3	36.6	43.3	3.4	6.6	20	73.3
45 Min after prescription, %	6.6	10.0	40	26.6	16.6	3.4	56.6	36.6	3.4	6.6	26.6	66.6
60 Min after prescription, %	26.6	20	33.3	26.6	3.4	0	20	56.6	23.3	16.6	40	43.3
*P* value	<0.001					<0.001				<0.001		

The mean O_2_ saturation at first was 97.50 ± 1.30 that at the last check changed to 96.33 ± 1.68. The trend of O_2_ saturation changes during sedation had significant decreases (P value = 0.000). None of the children suffered hypoxemia (O_2_ saturation under 90%). The mean RR at the onset was 22.23 ± 6.54 ; at the last visit it changed to 18.80 ± 4.81. Also the mean of HR at the onset was 112.46 ± 14.82 and at the last visit it changed to 103.90 ± 14.57. The trends of RR and HR changes had significant decreases (P value < 0.001). Moreover, the trends of systolic and diastolic BP changes also had significant decreases (P value < 0.001).

There was an overall side effect rate of 72.3%. The most common was euphoria (66.66%) followed by vertigo (6.7%); 27.7% did not present any side effects. All of the adverse effects resolved by observation only.

## 5. Discussion

Effectiveness of midazolam compared closely to other routine sedative agents such as propofol, fentanyl and ketamine (5, 6). Midazolam IM for temporary short-term pediatric sedation was safe and effective; the greatest sedative impact occurred 45 minutes after injection consistent with other investigations (3, 5, 6). Demographic characteristics such as age were not influential on the alteration of vital signs.

Psychological side effects such as hallucination and agitation have been commonly reported for benzodiazepines but euphoria with this high incidence has been reported rarely. One reason for this high incidence might be race (7-9). Previous studies have shown considerable alteration in vital signs as an adverse effect of midazolam; these changes have been temporary (3, 10). On the other hand, insufficient dose may not able to provide a deep sedation and further doses may increase the risk of serious side-effects (11, 12). Although, mentioned changes was dose dependent, it seems reasonable that the patient under sedation be observe closely. It seems that children who receive intramuscular midazolam may be susceptible to vital signs alterations. Further investigation with a control group and larger sample size and other forms of midazolam administration (such as rectal suppositories) is recommended.
